# Coordinated Cell Type–Specific Epigenetic Remodeling in Prefrontal Cortex Begins before Birth and Continues into Early Adulthood

**DOI:** 10.1371/journal.pgen.1003433

**Published:** 2013-04-11

**Authors:** Hennady P. Shulha, Iris Cheung, Yin Guo, Schahram Akbarian, Zhiping Weng

**Affiliations:** 1 Program in Bioinformatics and Integrative Biology, University of Massachusetts Medical School, Worcester, Massachusetts, United States of America; 2 Brudnick Neuropsychiatric Research Institute, University of Massachusetts Medical School, Worcester, Massachusetts, United States of America; 3 Departments of Psychiatry and Neuroscience, Mount Sinai School of Medicine, New York, New York, United States of America; Ludwig Institute for Cancer Research, University of California San Diego, United States of America

## Abstract

Development of prefrontal and other higher-order association cortices is associated with widespread changes in the cortical transcriptome, particularly during the transitions from prenatal to postnatal development, and from early infancy to later stages of childhood and early adulthood. However, the timing and longitudinal trajectories of neuronal gene expression programs during these periods remain unclear in part because of confounding effects of concomitantly occurring shifts in neuron-to-glia ratios. Here, we used cell type–specific chromatin sorting techniques for genome-wide profiling of a histone mark associated with transcriptional regulation—H3 with trimethylated lysine 4 (H3K4me3)—in neuronal chromatin from 31 subjects from the late gestational period to 80 years of age. H3K4me3 landscapes of prefrontal neurons were developmentally regulated at 1,157 loci, including 768 loci that were proximal to transcription start sites. Multiple algorithms consistently revealed that the overwhelming majority and perhaps all of developmentally regulated H3K4me3 peaks were on a unidirectional trajectory defined by either rapid gain or loss of histone methylation during the late prenatal period and the first year after birth, followed by similar changes but with progressively slower kinetics during early and later childhood and only minimal changes later in life. Developmentally downregulated H3K4me3 peaks in prefrontal neurons were enriched for Paired box (Pax) and multiple Signal Transducer and Activator of Transcription (STAT) motifs, which are known to promote glial differentiation. In contrast, H3K4me3 peaks subject to a progressive increase in maturing prefrontal neurons were enriched for activating protein-1 (AP-1) recognition elements that are commonly associated with activity-dependent regulation of neuronal gene expression. We uncovered a developmental program governing the remodeling of neuronal histone methylation landscapes in the prefrontal cortex from the late prenatal period to early adolescence, which is linked to cis-regulatory sequences around transcription start sites.

## Author Summary

Prolonged maturation of the human cerebral cortex, which extends into the third decade of life, is critical for proper development of executive functions such as higher-order problem-solving and complex cognition. Little is known about changes of post-mitotic neurons during this prolonged maturation period, including changes in epigenetic regulation, and more broadly, in genome organization and function. Such knowledge is critical for a deeper understanding of human development, cognitive abilities, and psychiatric diseases. Here, we identify 1,157 genomic loci in neuronal cells from the prefrontal cortex that show developmental changes in a chromatin mark, histone H3 trimethylated at lysine 4 (H3K4me3), which has been associated with regulation of gene expression. Interestingly, the overwhelming majority of these developmentally regulated H3K4me3 peaks were defined by rapid gain or loss of histone methylation during the late prenatal period and the first year after birth, followed by slower changes during early and later childhood and minimal changes thereafter. The genomic sequences showing these dynamic changes in H3K4me3 were enriched with distinct transcription factor motifs. Our findings suggest that there is highly regulated, pre-programmed remodeling of neuronal histone methylation landscapes in the human brain that begins before birth and continues into adolescence.

## Introduction

Prolonged maturation of the human cerebral cortex, which extends into the third decade of life, is critical for proper development of executive functions, such as higher order problem solving and complex cognition [1], [2]. Little is known about changes within the nuclei of post-mitotic neurons during this prolonged maturation period, including possible changes in epigenetic regulation of DNA and histone proteins. This lack of knowledge is remarkable given that neurogenesis and subsequent permanent exit from the cell cycle by newly generated cortical neurons takes place within the first 3–4 months of prenatal development. It is likely that dynamic changes in epigenetic regulation of neuronal gene expression extend far beyond the first trimester of pregnancy. For example, various periods of growth and differentiation in early and late childhood have been found to be followed by highly dynamic waves of pruning and remodeling of synapses and neuronal connectivity in the prefrontal cortex (PFC) and related areas [3].

Human postmortem brain studies have revealed robust transcriptional changes during the transition from the prenatal period to childhood and into adulthood, with broad implications for inhibitory and excitatory neurotransmission, myelination, metabolism, and various other cellular functions [4], [5]. Thus, it is reasonable to hypothesize that the developmental milestones of cortical neurons are linked to ‘pre-programmed’ changes in neuronal gene expression. These mechanisms are likely to be associated with the process of neural differentiation because genes controlling the process of cell division show a general decline in expression during prenatal development and infancy, while genes associated with synaptic functions and neurotransmission show an increased expression during the same period [5]. Likewise, adolescence and early adulthood (15–25 years) are accompanied by a transient increase in transcripts for energy metabolism as well as protein and lipid synthesis, in conjunction with a further decline in genes implicated in neurodevelopment and plasticity [6]. Each of these developmentally defined transcriptional changes simultaneously involves hundreds to thousands of genes distributed throughout the entire genome, raising the question of whether there is a coordinated unfolding of neuronal gene expression as the PFC matures.

Quantifying developmentally regulated changes in neuronal transcriptomes and epigenomes, however, is confounded by the rapidly occurring and dramatic shifts in cell composition during maturation of the human cerebral cortex. For example, there is a post-natal increase of several fold in the number of astrocytes generated by cell division from local precursors [7]. Although the prenatal cortical plate is overwhelmingly comprised of post-mitotic neurons, neuron-to-glia ratios in the mature primate cerebral cortex are in the range of 0.6 (human) to 1.7 (macaque) [8]. Developmental changes in the cortical transcriptome may largely reflect this underlying change in cell composition and, when explored in tissue homogenate, could mask cell type-specific regulation. To circumvent the potential confound of shifting cell compositions, we have developed the technique of sorting and separating neuronal and non-neuronal nuclei of human post-mortem PFC specimens for subsequent preparation of mono-nucleosomes (nucleosomes are the fundamental units of chromatin, composed of a histone core—the H2A/H2B/H3/H4 octamer—and 146 bp DNA wrapped around it) for genome-wide histone methylation mapping [9], [10]. For this work, we focused on histone H3 trimethylated at lysine 4 (H3K4me3), a histone modification sharply regulated around transcription start sites (TSS) and other regulatory sequences [11], [12]. The H3K4me3 mark broadly correlates with RNA polymerase II occupancy at sites of active gene expression [13], and is largely non-overlapping with promoter-associated DNA cytosine methylation and other repressive marks [14], which provides an additional component of transcriptional regulation [15], [16].

In an earlier study, we presented high-resolution maps of H3K4me3 in neuronal and non-neuronal nuclei collected from the PFC of 11 individuals ranging from 0.5 to 69 years [9]. We identified thousands of genes that showed highly enriched H3K4me3 levels in neuronal but not non-neuronal PFC chromatin. We also identified hundreds of genes (many function in developmental processes) with decreased H3K4me3 during the first year after birth. Here, building upon our earlier results, we present H3K4me3 maps of 36 human PFC specimens collected from the late prenatal period to 81 years of age, with an emphasis on comparing the H3K4me3 landscape in prenatal PFC with that of older ages. We present evidence that 1157 loci within the neuronal epigenome of the PFC are subject to ongoing remodeling of the local H3K4me3 landscape. This remodeling is defined by a unidirectional trajectory of progressive gain or loss of H3K4me3, with steeper changes in the late prenatal period until the first year after birth, and slower changes thereafter extending into the adolescence and adult period. The genes near these age-dependent H3K4me3 peaks showed similar temporal expression patterns, i.e., genes near regions with increasing H3K4me3 also show increasing expression as a function of age and vice versa. Furthermore, in neuronal chromatin, genes near developmentally up-regulated H3K4me3 peaks were enriched in various functional categories related to mature neurons. In contrast, non-neuronal chromatin was defined by a progressive H3K4me3 increase at transcription start sites (TSS) associated with myelination and other glial-related functions. Our results draw a connection between cis-regulators of chromatin structure and function as well as the molecular mechanisms governing the maturation of the human prefrontal cortex, starting with prenatal development and continuing into adolescence and beyond.

## Results

### Chromatin immunoprecipitation followed by deep sequencing

We analyzed 36 datasets of H3K4me3 chromatin immunoprecipitation followed by sequencing (ChIP-seq) of sorted nuclei from the human prefrontal cortex. Thirty-one of these datasets were for neuronal NeuN+ nuclei and as control, the other five datasets were for non-neuronal (NeuN–) nuclei. The neuronal (NeuN+) samples covered an age range from 34 gestational weeks (gw) to 81 years (yr), including three prenatal samples (C1–C3) and three infant samples (<1 yr old; C4–C6). The NeuN– samples spanned an age range of 40 gw to 69 yr, including one prenatal sample and one infant sample. Among these datasets, 16 neuronal samples (including the 3 prenatal) and two NeuN– samples were newly generated and sequenced for this study, and the remaining 18 datasets were taken from our previous publications [9], [10]. We also used one input dataset based on the NeuN+ cells of C28 [10] that had been previously generated, but for which no antibody was added though processed in the same way as the above ChIP-seq samples (Table 1).

**Table 1:** Samples used in this study and sequencing statistics.

Sample ID	Brain bank	Gender	Age	PMI	pH	Total reads (M)	Unique map (%)	% Unique map in promoter	Cause of death	Previously published
C1	UM-BTB	F	34 gw	9	6.55	12.3	82	35	Multiple congenital anomalies	no
C2	UM-BTB	F	39 gw	2	N/a	14.5	84	45	Unknown	no
C3	UM-BTB	F	40 gw	11	6.21	13.9	87	51	Respiratory insufficiency	no
C4	UM-BTB	M	0.5 yr	N.D.	6	6.7	85	67	Complications of prematurity	PNAS
C5	UM-BTB	M	0.6 yr	8	6.6	2.1	73	67	Positional asphyxia	PNAS
C6	UM-BTB	F	0.8 yr	8	6	4.7	80	58	Respiratory insufficiency	PNAS
C7	UM-BTB	M	1.3 yr	19	6.5	3	85	68	Drowning	PNAS
C8	UM-BTB	F	2.8 yr	12	6.5	6.3	78	51	Drowning	PNAS
C9	HBB	F	4 yr	N/a	6.17	11.9	74	69	Acute pneumonia	no
C10	UM-BTB	M	4.7 yr	17	6.5	3.8	79	63	Drowning	PNAS
C11	UM-BTB	M	8.8 yr	5	6.7	2.2	81	71	Cardiac arrhythmia	PNAS
C12	UM-BTB	M	14 yr	13	7	3.2	74	55	Accident	PNAS
C13	HBTC	M	17 yr	30.8	6.55	20.2	84	38	Hanging	no
C14	UCI/UCD	M	18 yr	22	6.97	12.8	84	55	Drowning	no
C15	HBTRC	M	19 yr	18.6	6.02	13.2	89	68	Pneumonia	no
C16	MPRC	M	20 yr	15	6.4	10.7	86	56	Gun shot to chest, homicide	AGP
C17	MPRC	M	23 yr	12	6.6	13.4	83	38	Unknown	no
C18	MPRC	M	24 yr	22	6.5	4.1	82	39	Gun shot to chest, suicide	AGP
C19	HBTRC	M	24 yr	21.3	6.29	18.4	80	38	Myocardial infarction	no
C20	HBTRC	M	26 yr	32.8	6.21	18.1	87	58	Heart Attack	no
C21	MPRC	F	28 yr	24	6.7	23.4	85	49	Congenital heart disease	AGP
C22	UCI/UCD	M	38 yr	16	6.1	7.6	66	71	Cardiac	AGP
C23	HBTRC	F	41 yr	14	6.62	12.7	81	24	Unknown	no
C24	MPRC	M	55 yr	17	6.9	6	82	51	Cardiac	AGP
C25	UCI/UCD	M	63 yr	24.5	6.88	8.4	90	70	Cardiac	no
C26	HBTRC	M	64 yr	28.2	6.23	17.5	81	24	Unknown	no
C27	UCI/UCD	F	68 yr	7	6.07	8.3	72	51	Respiratory failure	PNAS
C28	UCI/UCD	F	69 yr	40	6.3	4.6	83	62	Cardiac	PNAS
C29	MPRC	M	74 yr	12	6.65	3.1	83	60	Cardiac	no
C30	MPRC	F	74 yr	18.5	7.21	7.5	90	70	Cardiac	no
C31	UCI/UCD	M	81 yr	8	6.6	10.3	77	53	Cardiac	no
C28input	UCI/UCD	F	69 yr	40	6.3	15.9	77	4	Cardiac	AGP
C3-	UM-BTB	F	40 gw	11	6.21	13.0	81	22	Respiratory insufficiency	no
C32-	UM-BTB	M	0.6 yr	20	6.6	17.3	80	22	Unknown	no
C33-	HBTRC	M	19 yr	18.6	6.02	15.0	76	16	Pneumonia	AGP
C34-	HBTRC	M	24 yr	21.3	6.24	17.8	77	16	Myocardial infarction	AGP
C35-	UCI/UCD	F	69 yr	7.25	6.01	6.2	84	51	Multiple injuries	PNAS

Brain Banks: HBTRC - Harvard Brain Tissue Resource Center (Dr. Francine Benes); UM-BTB - University of Maryland Brain and Tissue Bank for Developmental Disorders (Dr. Ron Zielke); MPRC - Maryland Psychiatric Research Center (Dr. Rosalinda Roberts and Dr. Andree Lessard); UCI/UCD - University of California Irvine/Davis (Dr. Ted Jones and William E. Bunney Jr). AGP = Shulha et al. Archives of General Psychiatry 69:314–324 (2012); PNAS = Cheung et al., Proceedings National Acad Sci USA 107:8824–8829 (2010). gw = gestational week.

Samples were sequenced using Illumina GAII and a total of 390 million (M) 36-bpreads were obtained, where 318 M reads mapped to unique locations in the human genome (Table 1). The data are available at http://zlab.umassmed.edu/zlab/publications/ShulhaPLOSGen2013.html. On average, 50% uniquely mapped reads in ChIP-seq samples were in promoters (within 2 kb of the TSS) of RefSeq genes (http://www.ncbi.nlm.nih.gov/RefSeq/; 35,519 transcripts in total), and 4% reads of the input sample were in promoters, consistent with the prior knowledge that H3K4me3 is strongly enriched around TSS [17]. We used the MACS algorithm [18] to detect the genomic regions significantly enriched in H3K4me3 (called peaks) in each ChIP-seq sample compared with the input sample. We then constructed a pool of 47,281 neuronal peaks detected in at least one neuronal sample, and another pool of 42,693 peaks detected in at least one NeuN– sample. We performed further analysis on these two pools of peaks. For each peak, we counted the total number of reads in each sample, normalized by the total number of reads in that sample that mapped to within 2 kb of all RefSeq TSSs.

### Age-dependent neuronal H3K4me3 profiles in promoters

**Figure 1:**
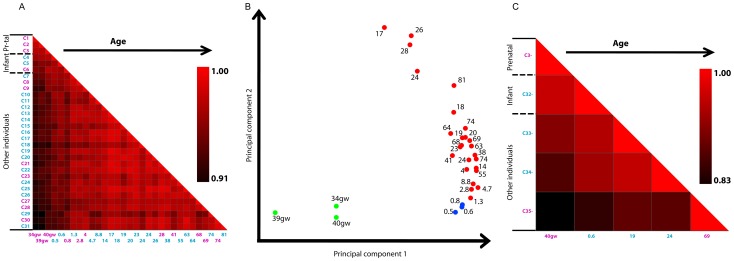
Prenatal age and early infancy are associated with transition of H3K4me3 landscapes in prefrontal cortex. A. Correlation of H3K4me3 occupancy in RefSeq promoters between all pairs of samples. Samples are sorted by age. The ages of the samples are listed on the x-axis in years (except where gw = week of gestation), with males in blue and females in pink. Note that the six youngest brains (prenatal and infant brains <1 year of age) are less strongly correlated with older brains (Pearson correlation coefficient R = 0.91–0.97), while brains ranging from 1.3 to 81 years of age mostly show very high (R = 0.94–0.99) between-sample correlations. B. Principal Component Analysis (PCA) analysis of differential H3K4me3 peaks for discovering similarity between samples. Prenatal samples are in green, infant samples in blue, and the remaining samples in red. Ages for prenatal samples are in gw and for all other samples in years. Each sample was compared with every other sample to calculate the number of peaks that are different, and the resulting 31 by 31 matrix was used as the input for the PCA analysis. The first principal component clearly separates prenatal samples from the other samples and the second component shows lower values for both prenatal and infant samples than for older samples. C. The same as A but for the five NeuN– samples included in this study.

To investigate promoter H3K4me3 occupancy profiles in neurons, we constructed a vector for each neuronal sample with 21,084 elements (the total number of unique RefSeq TSSs excluding chromosome Y), each element being the normalized number of reads in a RefSeq promoter. Then we computed the Pearson correlation coefficient for all pairs of samples and presented the results as a heatmap (Figure 1A). All 31 samples are highly similar, with correlations above 0.9. The most distinct samples are the three prenatal samples C1–C3, followed by the three infants C4–C6, as shown by the darker shades of the first six columns in Figure 1A. Thus, from the prenatal period to less than 1 year of age to older childhood and adulthood, there is an age-dependent progression of the genome-wide H3K4me3 profile in annotated promoter regions. These findings are consistent with observations we previously reported [9], [10] using much smaller cohorts and a narrower age range.

### Principal component analysis of neuronal H3K4me3 peaks

To determine how the overall population of H3K4me3 peaks (regardless of their association with any annotated TSS) differ across the neuronal samples, we performed principal components analysis on all H3K4me3 peaks as previously described [10]. Principal components analysis is a mathematical method that reduces the dimensionality of data, in our case, a 31 by 31 matrix for which each element is the number of H3K4me3 peaks found in one sample contrasted with another sample. The analysis identifies directions, called principal components, which maximize the variation in the data. Samples are then plotted along the principal components to reveal clusters. Figure 1B shows the 31 neuronal samples plotted against the first two principal components, which in combination account for 95% of the variation in our data. Strikingly, the first principal component separates the prenatal samples (in green) away from all other samples, and the second principal component separates the prenatal samples and the infant samples (in blue) from the remaining samples (in red). The third and fourth principal components do not further separate the samples older than 1 yr into subgroups (data not shown). In order to investigate whether the clear separation is due to the larger number of samples in the >1 yr group, we also performed PCA with 12 samples, 3 samples in each age range: gestational, 0–1 years, 3–14 years, and 15–25 years. The results looked very similar, namely the first two principal components could clearly distinguish the gestational and infant groups from the remaining samples (Figure S1). Thus, neurons in the prefrontal cortex undergo substantial changes in their H3K4me3 landscapes during the transition from the last 2 months of gestation to postnatal life, to a somewhat lesser degree during the first year after birth, and comparatively minor changes for the remainder of the lifespan. With the exception of the three prenatal samples who were all females, all other age groups comprised males and females. However, neither sample-to-sample Pearson correlation analysis of RefSeq promoters nor PCA analysis (Figure 1A–1C) showed any evidence for a role of gender in the age-dependent remodeling of neuronal and non-neuronal H3K4me3 landscapes in the PFC. Notably, a recent study in a cohort of 269 postmortem specimens collected across the lifespan reported developmentally regulated changes in gene expression and a consistent molecular architecture of the PFC across the human race [5]. Our histone methylation studies support this conclusion because each of our age groups of prenatal, infant, older children and adults included subjects from at least two races (information on race was available for 14 brains: 8 Caucasian and 6 African American).

### Genomic loci and genes with age-dependent regulation of H3K4me3

**Figure 2:**
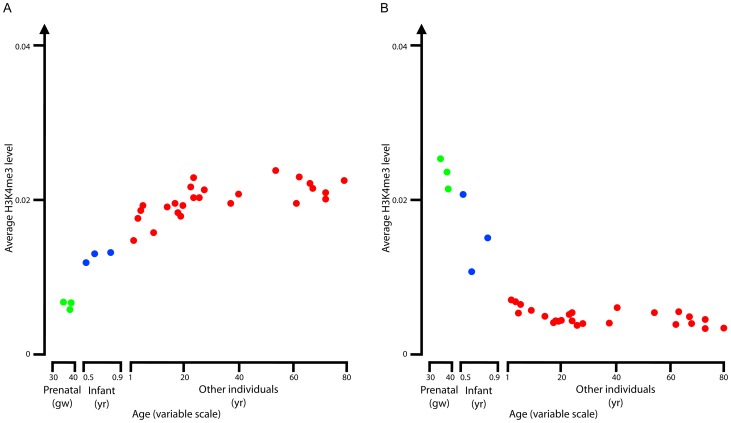
Averaged age profiles. Average age profiles for H3K4me3 level for the up (A) and down (B) peaks. Green corresponds to prenatal samples, blue for infant samples, and red for the remaining samples.

To identify H3K4me3 peaks subject to age-dependent regulation, we systematically compared the three prenatal samples with the 25 samples older than 1 year of age and found 742 (415) peaks with at least two-fold higher or lower levels in the prenatal samples (p-value<0.05; see Methods for details). We called these ‘down’ and ‘up’ peaks, reflecting the change of H3K4me3 upon aging (Table S1). We computed the average H3K4me3 levels for the up and down peaks in each sample. We observed a progressive increase (for the up peaks; Figure 2A) and decrease (for the down peaks; Figure 2B) as a function of age that continued at least for the first 10–20 years of life. Note that each of the three infant samples (<1 yr; blue dots in Figure 2A, 2B) was positioned in between the prenatal samples and the older child/adult samples although these three samples were not used to define the up and down peaks. Figure S2 uses boxplots to illustrate the distributions of H3K4me3 levels for the peaks within the ‘up’ and ‘down’ groups, respectively. It is clear that the variation among genes within each group is smaller than the difference between the two groups. From these results, we conclude that the PFC neuronal population undergoes a steady and continuous developmental remodeling of H3K4me3 peaks, starting during prenatal life and extending into the first childhood years.

**Figure 3:**
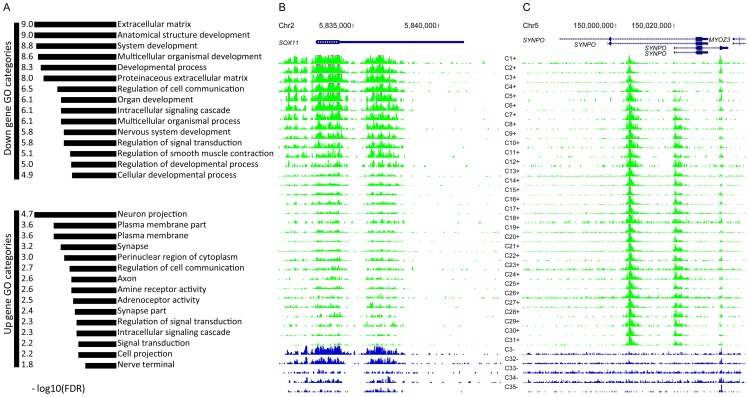
Top GO categories for genes with age-differential peaks. (A) Top 15 categories for up and down genes; (B) An example down gene; (C) An example up gene.

These up and down peaks were within 2 kb of the TSSs of 247 and 508 RefSeq genes respectively, and using the DAVID tool, we asked whether these two groups of genes were enriched in any gene ontology (GO) categories [19]. The 508 genes near the down peaks were enriched in 117 GO processes with a false discovery rate (FDR) of less than 5% (Table S2). Some of the most significant GO categories found in the down peaks included ‘anatomical structure’, ‘organ’, ‘systems’, ‘nervous system’ development (FDR ranges from 1.1e-9 to 1.6e-6), and many other processes of critical importance of early embryonic development (Figure 3A, Table S2). A representative example is the transcription factor SOX11 (SRY-related HMG-box) which among other functions, regulates neuronal differentiation during early development [20] (Figure 3B). The 247 genes near the up-peaks were enriched in 28 GO categories with FDR<5% (Table S2), and the most significant categories included neuron projection (FDR = 2.2e-5), synapse (FDR = 6.1e-4), and axon (FDR = 2.5e-3, Figure 3A). Thus, H3K4me3 levels near neuronal genes related to the function of mature neurons, including synaptic transmission and connectivity are up-regulated during the transition from pre- to post-natal life. A representative example is synaptopodin (*SYNPO*), encoding an actin-associated protein enriched in dendritic spines and postsynaptic densities [21], [22] (Figure 3C). The genes near the down peaks and genes near the up peaks were similarly enriched in 11 GO categories, mostly involving cell-cell communication and signal transduction function (colored blue in Table S2); however, distinct sets of genes led to the enrichment of each shared category.

Some of these genes contain multiple promoters that overlapped with multiple age-dependent H3K4me3 peaks. The first group contained eleven genes whose alternative promoters overlapped H3K4me3 peaks with opposing developmental changes (one up peak and one down peak). Some of these genes are essential for normal brain development, including *SHANK2*, encoding a synaptic scaffolding protein that when mutated is responsible for mono- or oligogenic causes of autism and other neurodevelopmental disease [23], [24]; *LFC/ARHGEF2*, encoding a Rho-specific guanine nucleotide exchange factor important for neurogenesis and dendrite spine morphology [25], [26]; and *PLEKHG5*, a Pleckstrin homology domain-containing gene that is responsible for an autosomal recessive form of motor neuron disease [27] (Table S3).There were also five genes each with two promoters subject to a developmental increase in H3K4me3, including the calcium sensor *CABP1*,which regulates voltage-gated Ca^2+^ channels and synaptic short term plasticity [28], and *SEPT9*, a member of the cytoskeleton-related septin family, which is responsible for hereditary neuropathic syndromes such as amyotrophic neuralgia [29] (Table S3).Furthermore, the third set of nine genes harbored multiple promoters that were subject to a coordinated developmental downregulation of H3K4me3, including the trans-membrane protein *NOTCH3*, which is essential for preventing premature death of young differentiating neurons [30] and responsible for some forms of vascular disease in the mature brain [31], the Wingless-Int (WNT) protein, *WNT7B*, which shows distinct regional expression patterns during human brain development, including progenitor cells and sub-layers of the developing cortical plate [32], and *DPYSL3*, which encodes for a dihydropyrimidinase-like protein important for neuronal differentiation and regulated by NMDA glutamate receptors [33] (Table S3).

Earlier studies reported that the process of differentiation from pluripotent stem cells was associated with dynamic changes in the shape of the H3K4me3 profile, due to spreading into neighboring nucleosomes in differentiated tissue, thereby resulting in broader peaks [34], [35]. To find out whether maturation of PFC is associated with similar changes in neuronal H3K4me3 landscapes, we determined for each subject numbers and proportion of peaks across six different length categories (from 500 bp to 5 kb). However, no age-dependent trends emerged, because peaks <1 kb in length comprised the large majority of the total pool of peaks, while longer peaks (>4 kb) contributed little (3% or less) to each sample (Table S4). This result, however, is unsurprising, because the marker used in our study for nuclei sorting (NeuN) specifically labels postmitotic neurons and excludes stem cells. Furthermore, in good agreement with the above studies [34], [35] reporting that genes specifically expressed in differentiated tissues show wider histone methylation peaks, the broadest peaks (>4 kb) in neurons in the present study also showed a highly significant enrichment of Gene Ontology categories defining neurons. These categories include *nervous system development*, *neurogenesis, neuron differentiation*, *axonogenesis*, *neuron projection*, *synapse*, *and postsynaptic density* among others (Table S5).

### Systematic detection of gene clusters with age-dependent H3K4me3 profiles

The up and down H3K4me3 peaks described above show roughly monotonic changes of H3K4me4 levels as a function of age (largely driven by rapidly changing H3K4me3 levels during the transitions from the prenatal period to infancy and from infancy to early childhood). To test whether our dataset harbors genomic loci with a different, or non-monotonic age profile, we performed k-means clustering, an unsupervised learning technique to separate all H3K4me3 peaks into five clusters (k = 5) so that peaks within each cluster have similar age profiles. In Figure S3, peaks were shown to be increased in Cluster 1 and decreased in Clusters 2 and 3 with age (with Cluster 2 showing a greater decrease than Cluster 3, but both clusters are defined by the largest shifts occurring within the first few years of life). Clusters 4 and 5 showed age-invariant H3K4me3 profiles. We also performed clustering using larger k values but the results were qualitatively the same, with additional clusters showing similar patterns. Therefore, consistent with the hypothesis-driven method described above for identifying the up and down peaks, unbiased k-means clustering again resulted in the two main patterns of age-dependent H3K4me3 changes, with some loci going monotonically up and other loci going monotonically down upon aging. Thus we concluded that the overwhelming majority of the developmentally regulated H3K4me3 changes in neuronal chromatin of the prefrontal cortex are unidirectional and monotonic, with the changes during the successive transitions from the prenatal period to early infancy and from infancy to later childhood ages being much more pronounced than any shifts that may occur later in life.

We also performed GO enrichment analysis on the genes near Cluster 1 and 2 peaks, and the results are shown in Table S6. Because the peaks in Clusters 1 and 2 match the up and down peaks defined in the previous section, the results of GO enrichment are highly consistent. Specifically, the genes near Cluster 1 peaks are enriched in developmental processes, with ‘anatomical structure’, ‘system’, ‘organ’ development, ‘neurogenesis’ (and many other GO categories in Table S2) again among the most significantly enriched, and the genes near Cluster 2 peaks are enriched for many neuron-related categories, including ‘axon’, ‘neuron projection’ and others.

### Expression levels of genes near age-dependent H3K4me3 peaks

**Figure 4:**
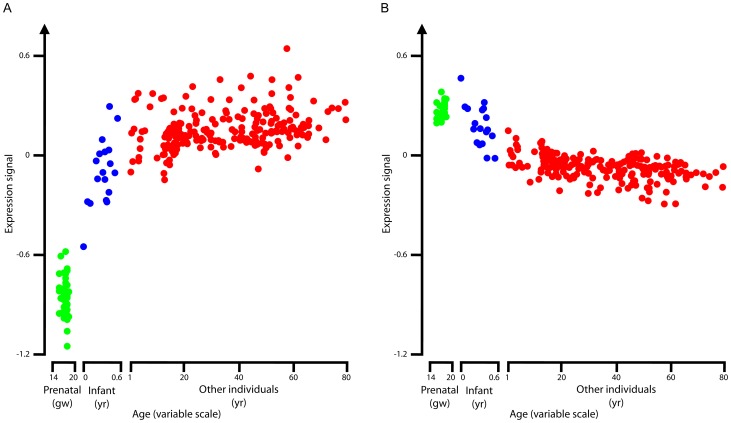
Average RNA expression levels. Average RNA expression levels for the genes near the up H3K4me3 peaks (A) and for the genes near the down H3K4me3 peaks (B). Expression data were taken from [5]. Each dot indicates one sample, for which the expression levels of the genes near the up (or down) H3K4me3 peaks were averaged. Color scheme is the same as for Figure 2.

H3K4me3 level is a good indicator of gene expression, and we wanted to further test whether the genes near the age-dependent H3K4me3 peaks show similar age-dependent expression patterns. Colantuoni *et al.* performed microarray experiments to assay the genome-wide transcription levels in the prefrontal cortex of 269 subjects spanning the majority of the human lifespan, including 38 prenatal samples (14–20 gws) and 18 infants (<1 yr) [5]. Using their data, we plotted, in Figure 4, the average expression levels of the 202 and 419 genes that are near our up and down H3K4me3 peaks which also assayed by Colantuoni et al. as a function of age. The expression signal as defined by Colantuoni et al. is the log2 density ratio of a particular sample over the reference sample produced by pooling all test samples [5]. It is clear that the genes that are near the up peaks are more highly expressed in older samples (Figure 4A) and the genes that are near the down peaks are expressed at lower levels in older samples (Figure 4B).

Similarly, we plotted the average expression patterns for the genes near each of the H3K4me3 ChIP-seq peaks in the five clusters determined by k-means clustering (Figure S4). Indeed, the average expression profile for genes in each cluster follows the same trend as the average H3K4me3 profile: genes near Cluster 1 peaks increased expression upon aging, genes near Cluster 2 and 3 peaks decreased expression upon aging with Cluster 2 showing a greater decrease than Cluster 3, and genes near Cluster 4 and 5 peaks show invariant expression across the age span.

Note that the prenatal gene expression data in Colantuoni et al. were for the 14–20 gw period [5], earlier than the stage of our H3K4me3 data (34–40 gw). They reported four groups of genes that showed significant changes in expression across age: genes that increased expression in both prenatal and infant stages (up-up genes), genes that decreased expression in both stages (down-down genes), genes that increased expression during the prenatal stage and then decreased expression during the infant stage (up-down genes), and genes that decreased expression during the prenatal stage and then increased expression during the infant stage (down-up genes). We plotted the H3K4me3 profiles for these four groups of genes (Figure S5). Indeed, both the up-up and down-up genes show a monotonic increasing H3K4me3 pattern (the left two panels of Figure S5), and both the down-down and up-down genes show a monotonic decreasing H3K4me3 pattern (the right two panels of Figure S5) However, the changes in these patterns are not as pronounced as our up and down H3K4me3 peaks (Figure 2 and Figure S3). Detailed examination of the expression patterns in Figure 2 of Colantuoni et al. indicates that the reversal of the expression patterns for the down-up and up-down genes occurs at around 19 gw. Because our H3K4me3 data are for later time points (34–40 gw), our two H3K4me3 patterns are consistent with the four expression patterns by Colantuoni *et al*.

### DNA methylation near age-dependent H3K4me3 peaks

In the human cerebral cortex, the genome-wide distribution of H3K4me3 is largely anti-correlated with methyl-cytosine densities [14] and many genes in the genome show a robust DNA methylation increase in CpG dense sequences, including those residing in proximal promoters [36], [37]. Using the datasets from [37], prenatal to postnatal changes in methyl-CpG densities in cortical tissue homogenates were available for 394 (744) CpGs that were in the promoters of genes subject to developmental H3K4me3 up- (down-) regulation in our study on PFC neurons (Table S7 and Table S8). Much higher percentages of the CpGs (59.9% and 60.1% respectively) were in the promoters of the genes near the up or down H3K4me3 peaks than the remaining 26,446 CpGs (42.0%) which show significantly different (FDR<0.05) methylation levels between prenatal samples and samples older than 1 yr. Moreover, 15.7% of CpGs associated with an *increase* in H3K4me3 during development showed a significant decrease in methyl-CpG levels (FDR<0.05), while only 6.2% genes associated with *declining* H3K4me3 levels also showed a significant decrease in methyl-CpG levels (Table S9). Thus, there is a significant anti-correlation between the age-dependent change of H3K4me3 levels and the age-dependent change in DNA methylation levels (p-value = 7.0e-8; hypergeometric test). These results are robust regardless of the cutoff for calling a DNA methylation as significantly different between prenatal samples and samples older than 1 yr (the lower half of Table S9 shows the results for the cutoff of FDR<1e-10).Examples of genes with significant and opposing changes in H3K4me3 and DNA methylation include *ARC, NR4A1* and other transcription factors with critical roles in synaptic plasticity, learning and memory [38], [39], *ANK3*, a psychiatric susceptibility genes encoding a synaptic scaffolding proteins [40], and ligand-gated ion channels including the GABA_A_ receptor subunit GABRD, which has been linked to mood and seizure disorders [41], [42].

### Transcription factors motifs enriched in age-dependent H3K4me3 peaks

We also tested whether the sequence motifs of any transcription factors (TF) are enriched in the up or down peaks using the Clover algorithm [43] with all TF binding motifs in the TRANSFAC database [44]. We used two types of background sequences, one was generated by shuffling the sequences of the up or down peaks while preserving dinucleotide frequencies, and the other was the H3K4me3 peaks that did not vary their intensities across age (peaks in Clusters 4 and 5). Table S10 lists the motifs that are significantly enriched in the up or down peaks according to both types of backgrounds (FDR<0.05). Among them, the motif ofAP-1 is significantly enriched among the up peaks. AP-1 (heterodimer of c-Jun and c-Fos) is a classical early response transcription factor and master regulator of the axonal response in neurons. It also functions as a negative regulator of myelination in Schwann cells (SCs) and is strongly reactivated in SCs upon axonal injury [45]. The Pax motif is highly enriched in the down peaks. Among the Pax family of transcription factors, Pax2 and Pax8 specify GABAergic cell fate [46], Pax6 is the master regulator of the visual system [47], and Pax8 is important for hindbrain neurons [48]. The motif of Rp58 is also enriched in the down peaks, and Rp58 is recently shown to be essential for the patterning of the cerebellum and for the development glutamatergic and GABAergic neurons [49]. We also noticed significant enrichment for Signal Transducer and Activator of Transcription (STAT) motifs in the down H3K4me3 peaks from PFC neurons, which agrees well with the finding that STAT-dependent signaling pathways primarily promote astrocytic and other non-neuronal differentiation in the developing cortical plate [50]–[52].

### Age-dependent H3K4me3 profiles in NeuN– nuclei

To further explore which, if any, of the above mentioned developmental H3K4me3 changes are specific to PFC neurons, we explored H3K4me3 landscapes in non-neuronal (NeuN–) nuclei obtained from one prenatal, one infant and three adult specimens (Table 1). Similar to the findings in the 31 neuronal samples described in Figure 1A, computation of Pearson correlation coefficient for all five pairs of NeuN– samples revealed higher correlations between samples of similar age (Figure 1C). The smaller number of NeuN– samples prevented us from using the same approach for identifying up and down H3K4me3 peaks as for NeuN+ samples, i.e., directly comparing prenatal samples with samples older than 1 yr, due to the lack of statistical power. Instead, we used the k-means clustering algorithm to identify 2224 TSS-proximal peaks (within 2 Kb of a TSS) subject to decline upon aging, and 785 TSS-proximal peaks that increased upon aging. The genes whose TSSs are proximal to the NeuN– H3K4me3-down peaks partially overlapped with the genes that are proximal to the NeuN+ H3K4me3-down peaks (1889;241;392 for the NeuN– unique, shared, and NeuN+ unique genes; chi-square test p-value<2.2e-16). Yet, these two sets of H3K4me3-down genes fall into similar GO categories (compare Tables S6 and S11). We determined the GO categories that each set of genes was enriched in and observed a strong correlation between the enrichment scores of the two sets of GO categories (Pearson correlation coefficient R = 0.70; p-value<1e-6). A GO category was included in the calculation if the FDR was less than 0.85 for either gene set and the enrichment score was defined as the –log_10_(FDR). Both gene sets were highly enriched in five GO categories related to development (multicellular organismal development, systems development, developmental process, anatomical structure development, and nervous system development). These results indicate that similar functional pathways are pruned epigenetically between neuronal and non-neuronal cells even though the genes that belong to these pathways differ between the two cell types.

Similar analysis for H3K4me3-up genes revealed a different picture. The genes whose TSSs are proximal to the NeuN– H3K4me3-up peaks overlapped partially with the genes that are proximal to the NeuN+ H3K4me3-up peaks (675;83;615 for the NeuN– unique, shared, and NeuN+ unique genes; chi-square test p-value<2.2e-16). However, these two sets of genes were enriched in mostly non-overlapping GO categories (compare Tables S6 and S11). The Pearson correlation coefficient between the enrichment scores of the two sets of GO categories was –0.11 (p-value = 0.24). The most enriched GO categories for NeuN– H3K4me3-up genes included compact myelin (FDR = 0.0029) and myelin sheath (FDR = 9.0e-5), consistent with one important function of glial cells, which make up the vast majority of NeuN– cells, and these GO categories were not enriched in any of the other three groups of genes (NeuN+ H3K4me3-up, NeuN+ H3K4me3-down, or NeuN– H3K4me3-down). On the other hand, the NeuN+ H3K4me3-up genes were more enriched in axon, neuron projection, and signal transduction (FDR = 0.0013–0.0052) than the NeuN– H3K4me3-up genes. We conclude that while both neuronal and nonneuronal cells undergo a major remarking of TSS-associated histone methylation during PFC development and maturation, different areas of the genome are affected, depending on cell type.

## Discussion

The present study provides detailed analyses into the developmental regulation of a transcriptional mark, H3K4me3, in neuronal and non-neuronal chromatin during the extended course of PFC maturation. There were 1157 loci, including 768 TSS-proximal and many other regulatory sequences that showed evidence for the developmental remodeling of H3K4me3. Strikingly, these peaks showed similar kinetics as defined by an unidirectional course with the largest decline or increase occurring within the first 1–2 years of postnatal life, followed by a gradual slowing of age-related changes that apparently continue at least until early adolescence or even beyond. We show that these developmentally regulated H3K4me3 peaks at transcription start sites are associated with age-related changes in the expression of the corresponding RNA. We further showed that a subset of regulatory motifs, including Pax and AP-1 transcription factor recognition sites, are overrepresented among the developmental regulated peaks showing a decrease (Pax, Stat), or increase (AP-1) respectively, during the course of PFC maturation. Finally, the developmental remodeling of H3K4me3 landscapes in PFC is cell type specific. Collectively, our results draw a connection between cis-regulators of chromatin structure and function and the molecular mechanisms governing maturation of the human prefrontal cortex.

The findings presented here, when taken in conjunction with studies exploring developmental changes in DNA methylation [36], [37] and gene expression across the lifespan of the human PFC [5], paint a picture in which immature PFC, during the weeks and months preceding and following birth, undergoes a larger scale reprogramming of transcriptomes and promoter-associated epigenetic regulators, including promoter-bound DNA and histone methylation. This reprogramming involves hundreds of TSS that define cellular functions that are either characteristics of differentiated neurons and glia (e.g. synaptic transmission, myelination) or functions related to earlier stages of development (e.g. neurogenesis, nervous system development) that decline as the PFC matures. By charting a developmental map for the neuronal and non-neuronal constituents of the PFC separately, the present study and our earlier studies [9], [10] largely extends the previous work on tissue homogenate that allows for limited data interpretation due to age-related shifts in neuron-to-glia ratios and other confounding factors. The present study further emphasizes the prenatal stage and shows that developmental remodeling of TSS-associated histone methylation in PFC neurons rapidly changes in prenatal and infant stages but continues at a slower pace deep into the second decade of life.

Our study faces several important limitations. Given that no technique with single cell resolution exists to map histone methylation levels at specific loci, our assays by design only inform about H3K4me3 profiles averaged across millions of cell type-specific nuclei that are required for the ChIP-seq assays. Thus, the cell population-based developmental kinetics of H3K4me3 with the simple and unidirectional exponential curves, as presented here, leave open whether individual PFC neurons would show a more dynamic interplay between H3K4 methylation and demethylation. Furthermore, our prenatal specimens were limited to the mid- and late stages of the third trimester, and it remains possible that brains of an earlier prenatal age could show a more complex or multi-layered regulation of the H3K4me3 marks, compared to what is reported here. For example, there are four groups of genes reported by Colantuoni et al., who compared RNA expression at earlier stages of gestation (14–20^th^ week of pregnancy) with those of infant and older brains [5]. Nonetheless, these four groups of genes could be recognized by their age-dependent H3K4me3 profiles in our study (Figure S5), reaffirming the view that these gene expression networks are co-regulated both on the level of the transcriptome and the epigenome.

The present study suggests that the developmental remodeling of TSS-associated histone methylation in PFC neurons in the weeks and months before and after birth continues at a slower pace deep into the second decade of life. Presently, nothing is known about the molecular ‘clocks’ or ‘pacemakers’ that orchestrate such steadfast remodeling of TSS-associated H3K4me3 during the first 10–20 years of life and, to the best of our knowledge, these phenomena await further investigation in laboratory animals. Indeed, a recent H3K4me3 ChIP-seq study in whole tissue of four macaque PFC specimens found evidence for histone methylation changes during the course of maturation and aging [53]. Insights into these mechanisms bear great promise for a better understanding of normal development and the pathophysiology of schizophrenia and autism and other neurodevelopmental disorders associated with DNA and histone methylation changes in the PFC [10], [54]–[58]. To this end, it is interesting that AP-1 transcription factor motifs are enriched in H3K4me3 peaks that are upregulated during the course of PF maturation. Of note, antipsychotic drug treatment administered over the course of 2–3 weeks resulted in lasting increases of AP-1 protein and transcript in rat rostromedial cortex (considered the functional homologue to primate PFC) [59]. Because AP-1 in PFC and other brain regions is highly regulated by neuronal activity [60], up-regulation of AP-1 expression and AP-1 mediated transcriptional activity, either during normal development or in the context of psychopharmacological treatments, could serve as one of the molecular drivers for the regulation of H3K4 trimethylation at AP-1 bound promoters and other active TSS in the mature PFC.

## Methods

All postmortem tissue work was done in compliance with the Institutional Review Board regulations of the University of Massachusetts Medical School and the Mount Sinai School of Medicine. Freshly frozen (never fixed) tissues from the rostral prefrontal cortex of subjects ranging in age from the 34^th^ week of gestation to 81 years, was provided by four independent brain banks (Table 1). Tissue aliquots (200–500 mg/subject) were extracted in hypotonic lysis buffer, purified by ultracentrifugation and resuspended in 1x PBS, immunotagged with anti-neuronal nucleus (anti-NeuN, Millipore) antibody and sorted into NeuN+ and NeuN– fractions using a FACSVantage SE flow cytometer, as described [61], [62].

Mononucleosomal preparations from at least 1×10^6^ sorted nuclei were prepared for subsequent chromatin immunoprecipitation with anti-H3K4me3 antibody (Upstate/Millipore), and ChIP-seq libraries prepared from the immunoprecipitated DNA by blunt-ending, A-tailing and ligation to adaptors (Genomic Adaptor Oligo Mix, Illumina) and PCR amplification and sequencing on an Illumina Genome Analyzer II platform, as described [9], [63].

All of our sequencing libraries contained single-end 36-bp reads and we mapped them to the human genome with Bowtie (version 0.11.3) [64]. We allowed up to one mismatch and mapped all sequences to the gender appropriate genome HG19. Reads that mapped to multiple locations were discarded. Unique mappers constitute 66–90% of all reads. Detailed statistics is presented in Table 1. As previously reported, H3K4me3 levels at promoters did not show correlations with postmortem interval and tissue pH [9], [65]. Critical ChIP-seq parameters, including the proportion of uniquely mappable sequence tags, and the percentage of uniquely mappable sequences at gene promoters were very similar between samples from the four brain banks, without significant differences (Table 1) (%uniquely mappable, % uniquely mappable at promoters: Harvard Brain Tissue Resource Center (HBTRC): 82.3 ± 4.9, 45.3 ± 19.3; Maryland Psychiatric Research Center (MPRC) 84.4 ± 2.9; 51.9 ± 11.4; University of California at Irvine/Davis (UCI/UCD) 78.7 ± 8.7; 60.3 ± 8.7; University of Maryland Brain and Tissue Bank for Developmental Disorders (UM-BTB),80.7 ± 4.5; 58.2 ± 10.5).

To construct the heatmap in Figure 1, we calculated Pearson correlation coefficients for each pair of samples. We took the genomic coordinates of all TSSs (except chrY) from RefSeq and expanded them in both directions by 2 kb. If some regions overlapped with each other, we merged them together. For each of these 21,084 non-overlapping regions, we computed the total number of mapped H3K4me3 ChIP-seq reads and normalized by size of the region. The resulting read densities were used to compute Pearson correlation coefficients.

The MACS software (version 1.3.5) [18] was used to identify statistically enriched H3K4me3 regions (called H3K4me3 peaks or peaks in short). We contrasted each sample against the input sample using bw = 230; tsize = 36 and default values for the remaining parameters in MACS.

Principal Component Analysis was performed on a matrix that contains peaks unique to each sample. Each sample was compared against every other sample using the MACS software with parameters mentioned above. The H3K4me3 peaks thus obtained were further filtered using the following criteria: (1) MACS p-value must be less than 1e-20, (2) read density ratio between the two samples must be greater than 4, and (3) normalized read density must be greater than 0.005.

To search for age-dependent H3K4me3 peaks, all peaks from NeuN+ samples were combined and overlapping peaks merged, resulting in 47,281 peaks. The 742 down peaks (≥1 k bp) were defined as: (1) the average read density in prenatal samples must be greater than or equal to 0.01, (2) the ratio of average read density of prenatal samples and the 25 samples older than 1 year must be greater than or equal to 2, and (3) the t-test p-value for comparing the three prenatal samples with the 25 samples older than 1 year must be less than or equal to 0.05. The reciprocal criteria were used for defining the 415 up peaks.

We performed k-means clustering on the age profiles of all H3K4me3 peaks. We limited our calculations to regions that were ≥1 k bp and had an average H3K4me3 read density ≥0.01 in prenatal samples or in the 25 samples older than 1 year. The small number of peaks in chrY was excluded from this analysis. This resulted in 14,708 regions that were further normalized by the strongest signal (across all samples) for each region. We then used the “k-means” procedure from the R software with 5 clusters. We averaged the H3K4me3 levels across the regions in each cluster and plotted the resulting average H3K4me3 profile for each cluster in Figure S3. We used the same approach to perform k-means clustering for NeuN– samples.

We used the DAVID web-server for detecting enriched Gene Ontology categories. For each set of H3K4me3 peaks, genes whose TSSs were within 2 k bp of an H3K4me3 peak are included in the analysis. We used false discovery rate (FDR) to quantify statistical significance, because it accounts for multiple testing correction.

To compare our H3K4me3 data with DNA methylation [37] we downloaded the data from the author's website (http://braincloud.jhmi.edu/downloads.htm).The dataset contains methylation level for CGs in a set of gene promoters. CGs in every gene that matched our proximal H3K4me3 peaks were analyzed for age-dependent changes (Tables S7 and S8). We performed a t-test for every gene, comparing all prenatal samples with all samples older than 1 year, and computed false discovery rate (FDR) after multiple testing correction. Cases with significant DNA methylation changes (using two cutoffs, FDR<0.05 or FDR<1e-10) were used to establish relationship with H3K4me3 (Table S9).

## Supporting Information

Figure S1 Principal component analysis of differential H3K4me3 peaks for discovering similarity between samples. Similar to Figure 1B, but with three samples in each age group: gestational, 0–1 years, 3–14 years, and 15–25 years. (A) The oldest group includes ages: 17, 18 and 24 yrs. (B) The oldest group includes ages: 19, 20 and 23 yrs.
(PDF)

Figure S2 Box plots showing the distributions of H3K4me3 levels of up genes (A) and down genes (B) in each sample, in correspondence with Figure 2. The bar in the box indicates median; the top and bottom edges of the box indicate the 25^th^ and 75^th^ percentiles; and the whiskers indicate the 10^th^ and 90^th^ percentiles.
(PDF)

Figure S3 Average age profiles for H3K4me3 level for five clusters obtained by k-means clustering. (A) Cluster 1; (B) Cluster 2; (C) Cluster 3; (D) Cluster 4; (E) Cluster 5.
(PDF)

Figure S4 Average age profiles for expression level for five clusters obtained by k-means clustering for H3K4me3. (A) Cluster 1; (B) Cluster 2; (C) Cluster 3; (D) Cluster 4; (E) Cluster 5.
(PDF)

Figure S5 Average age profiles for H3K4me3 level for four groups of genes defined by [5]: (A) the genes that increased expression in both prenatal and infant stages (up-up genes), (B) the genes that increased expression during the prenatal stage and then decreased expression during the infant stage (up-down genes), (C) the genes that decreased expression during the prenatal stage and then increased expression during the infant stage (down-up genes), and (D) the genes that decreased expression in both stages (down-down genes).
(PDF)

Table S1 The up (A) and down (B) H3K4me3 peaks. Numbers of TSS-proximal peaks and genes are different as several peaks can be located near the same gene and vice versa.
(XLSX)

Table S2 GO categories enriched in the genes whose TSS are within 2 kb of age-differential H3K4me3 peaks, with one sheet for up gene sets and another sheet for down gene sets. GO categories that are enriched in both down and up gene sets are colored in blue.
(XLSX)

Table S3 Genes with H3K4me3 regulation at alternative promoters (spaced up to 100 kb apart).
(XLS)

Table S4 Number and proportion of H4K4me3 peaks extending over >500 bp, >1 kb, >2 kb, >3 kb, >4 kb, and more than 5 kb.
(XLS)

Table S5 Gene ontology enrichments for H3K4me3 peaks >4 kb in nucleosomal preparations of NeuN+ PFC nuclei.
(XLS)

Table S6 GO categories enriched in the genes whose TSS are within 2 kb of age-differential H3K4me3 peaks for Cluster 1 and Cluster 2 derived from k-means clustering. GO categories that are enriched in both clusters are colored in blue.
(XLSX)

Table S7 CpG methylation levels at promoters of genes subject to developmental up-regulation of H3K4me3 levels in PFC neurons.
(XLS)

Table S8 CpG methylation levels at promoters of genes subject to developmental down-regulation of H3K4me3 levels in PFC neurons.
(XLS)

Table S9 Statistics on the CpGs that are in promoters of the genes near developmentally up- and down-regulated H3K4me3 peaks in PFC neurons that also show a statistically significant change in DNA CpG methylation between prenatal samples and samples older than 1 year. Two tables are provided for two cutoffs that define statistical change of methylation levels: top for FDR<0.05 and bottom for FDR<1e-10.
(XLSX)

Table S10 The transcription factor motifs enriched in the up or down H3K4me3 peaks.
(XLSX)

Table S11 GO categories enriched in the NeuN– genes whose TSS are within 2 kb of age-differential H3K4me3 peaks for Clusters corresponding to up and down groups derived from k-means clustering.
(XLSX)
