# Supplementary figures and images for: Coordinated Cell Type–Specific Epigenetic Remodeling in Prefrontal Cortex Begins before Birth and Continues into Early Adulthood
Source: PLoS Genet. 2013 Apr 11;9(4):e1003433. doi: 10.1371/journal.pgen.1003433 (PMC3623761; doi:10.1371/journal.pgen.1003433)

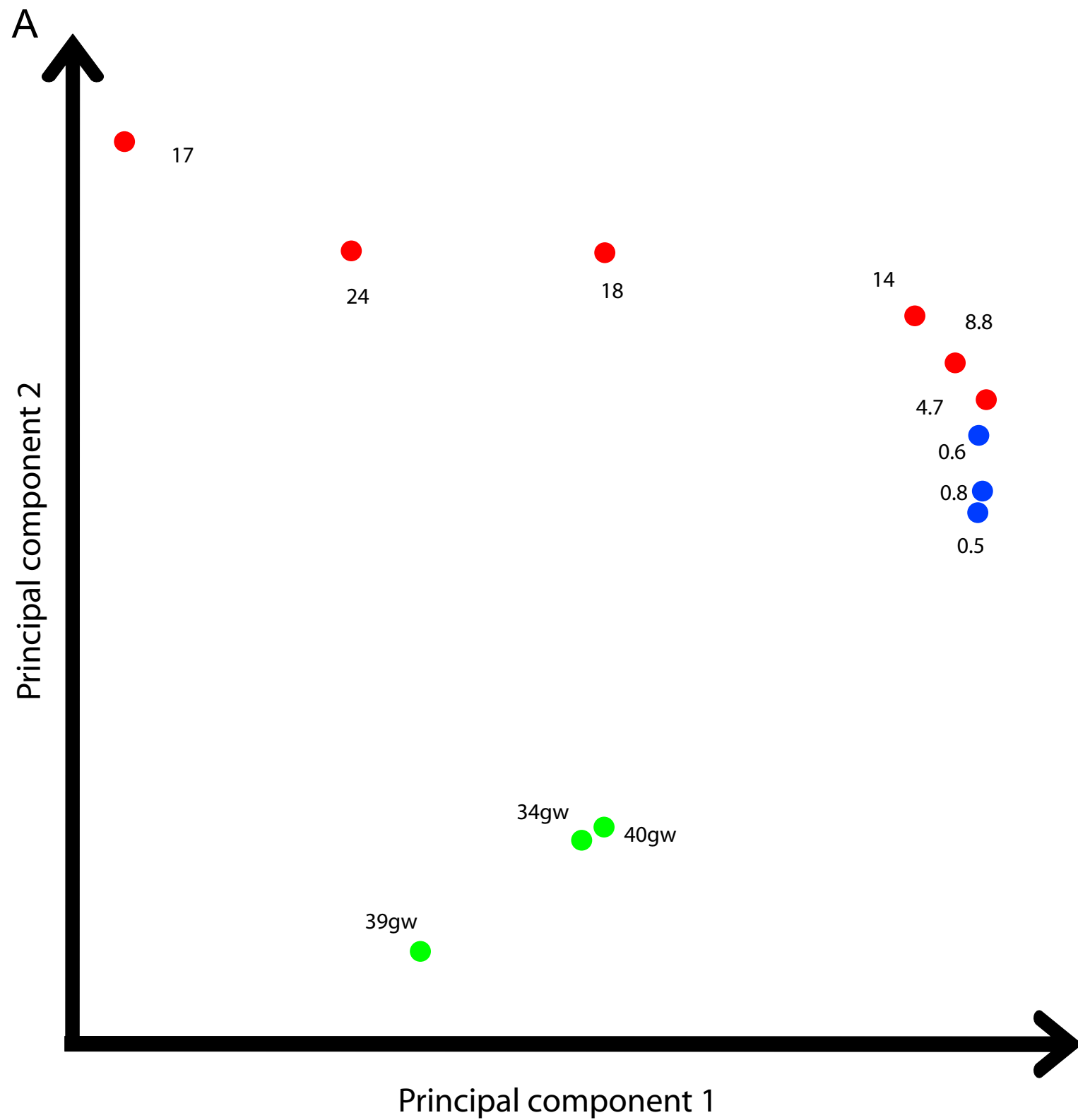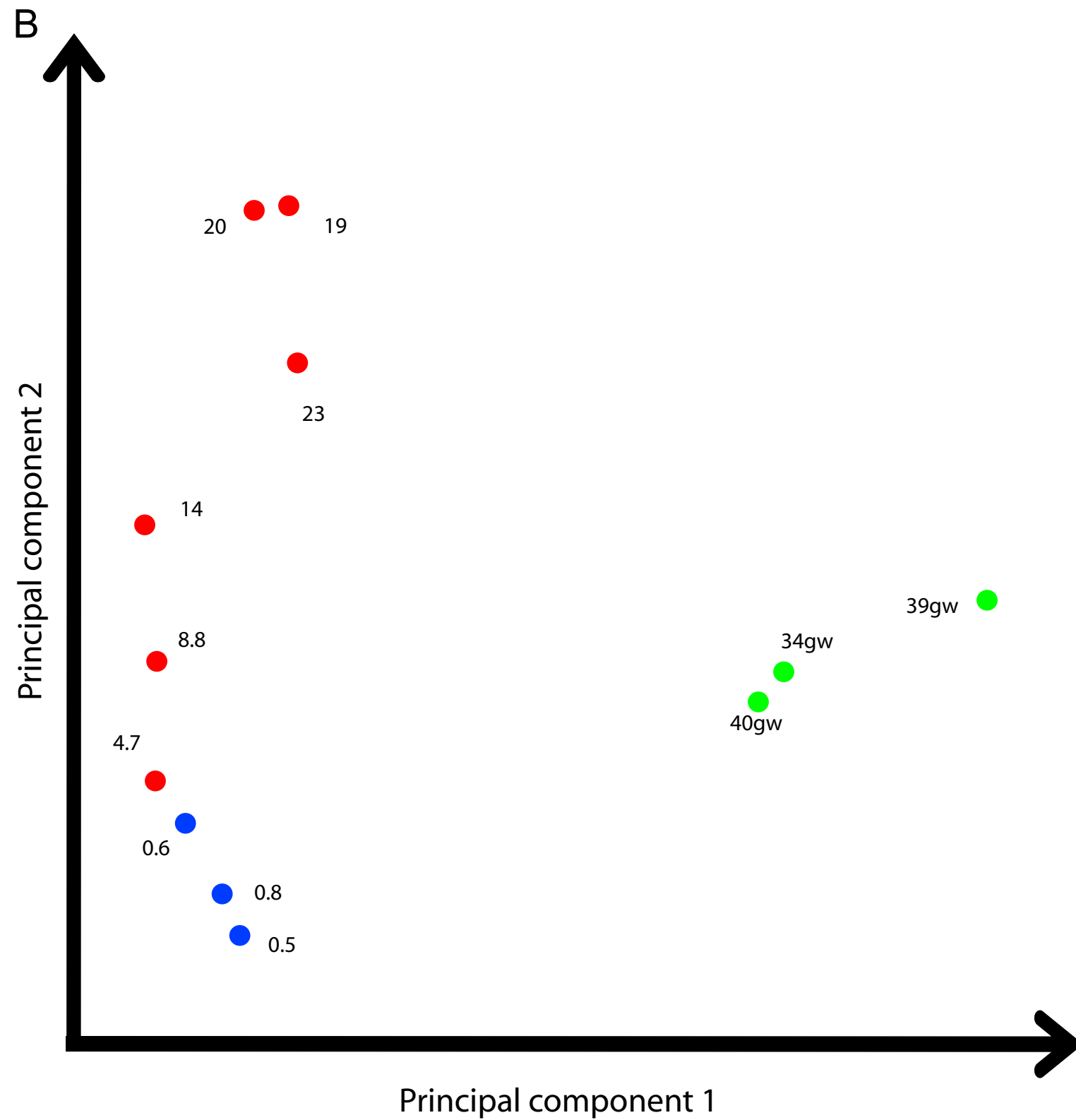

Supplement: Figure S1 — Principal component analysis of differential H3K4me3 peaks for discovering similarity between samples. Similar to Figure 1B, but with three samples in each age group: gestational, 0–1 years, 3–14 years, and 15–25 years. (A) The oldest group includes ages: 17, 18 and 24 yrs. (B) The oldest group includes ages: 19, 20 and 23 yrs. (PDF) [file pgen.1003433.s001.pdf]

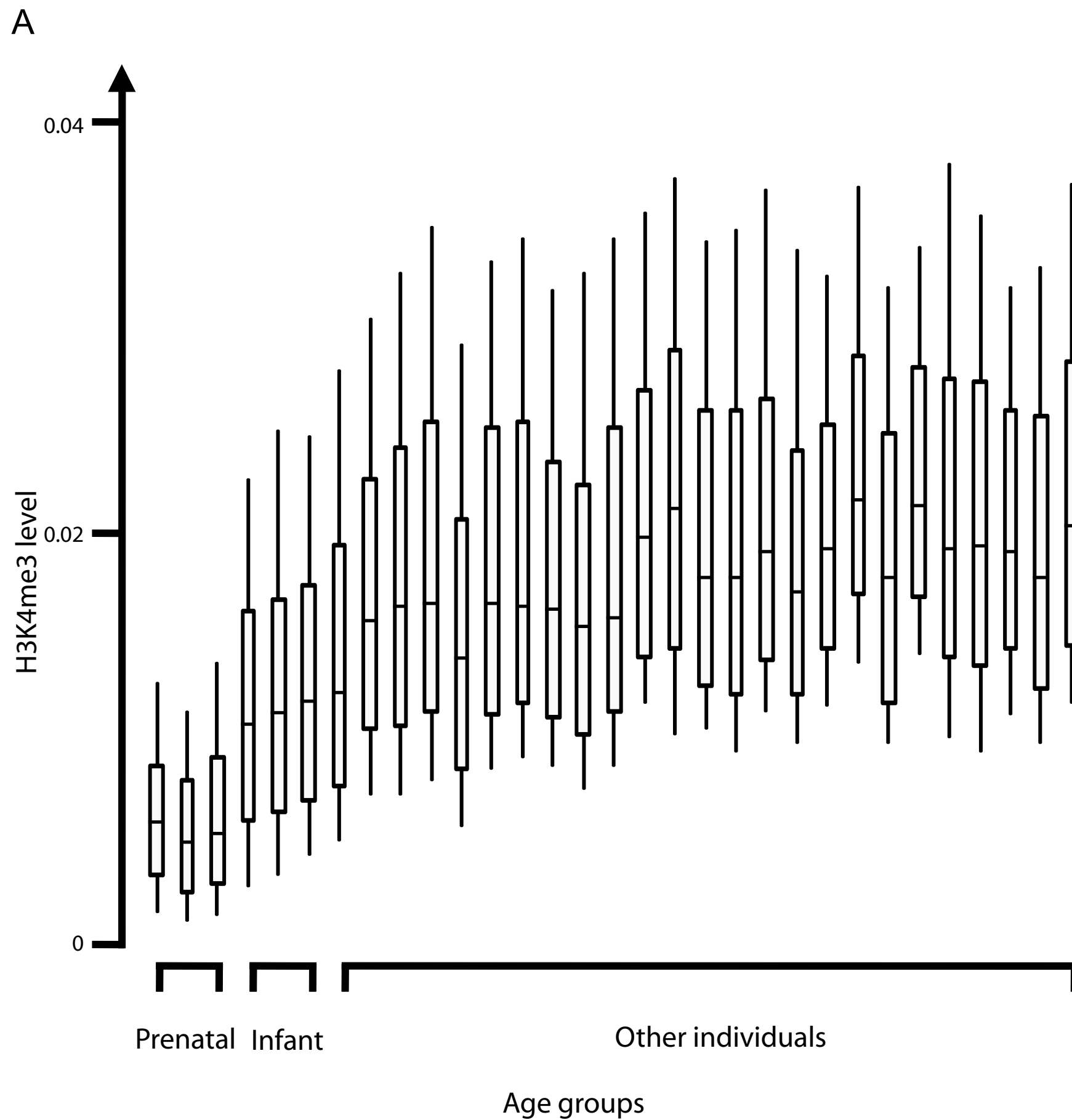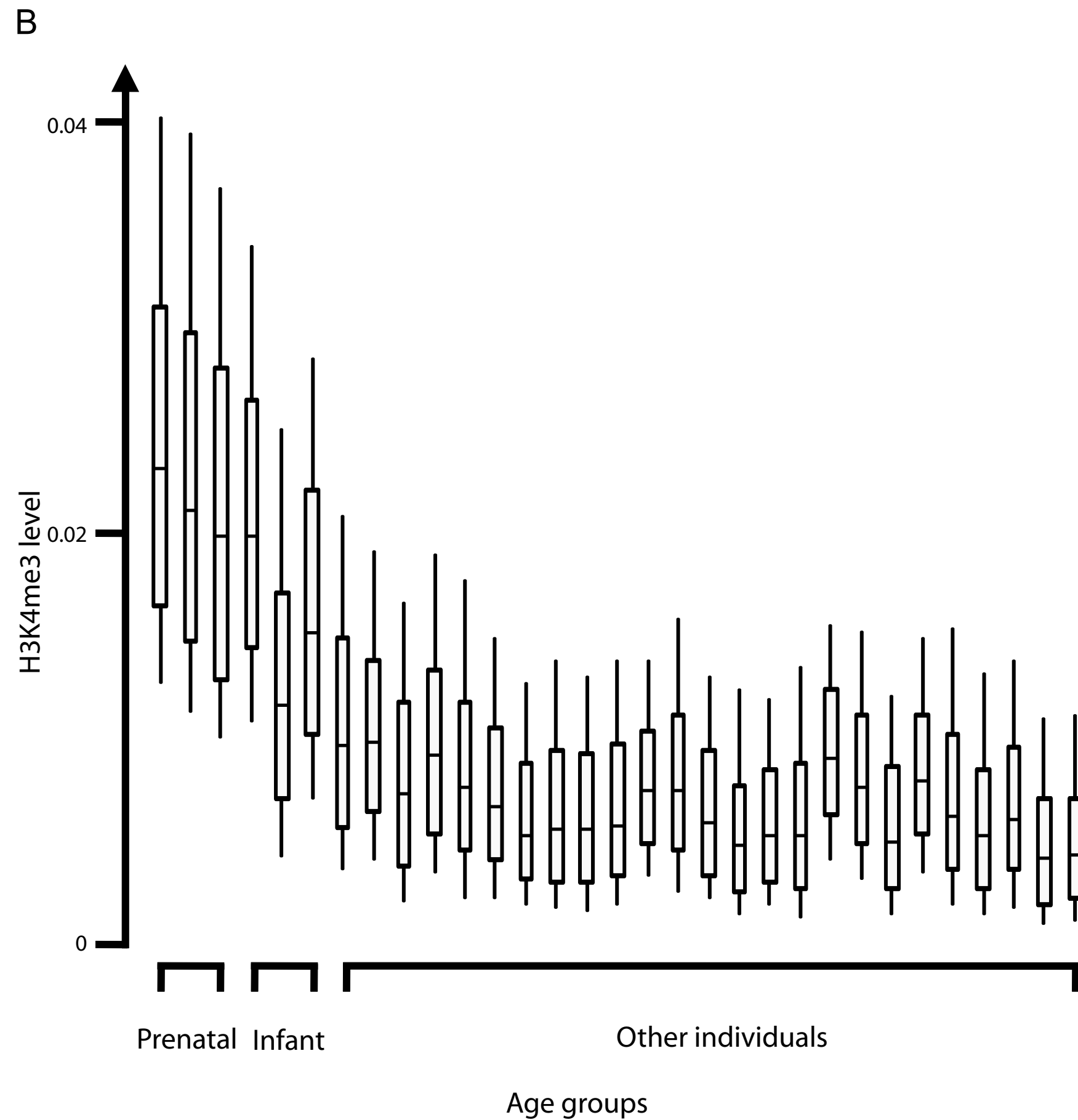

Supplement: Figure S2 — Box plots showing the distributions of H3K4me3 levels of up genes (A) and down genes (B) in each sample, in correspondence with Figure 2. The bar in the box indicates median; the top and bottom edges of the box indicate the 25th and 75th percentiles; and the whiskers indicate the 10th and 90th percentiles. (PDF) [file pgen.1003433.s002.pdf]

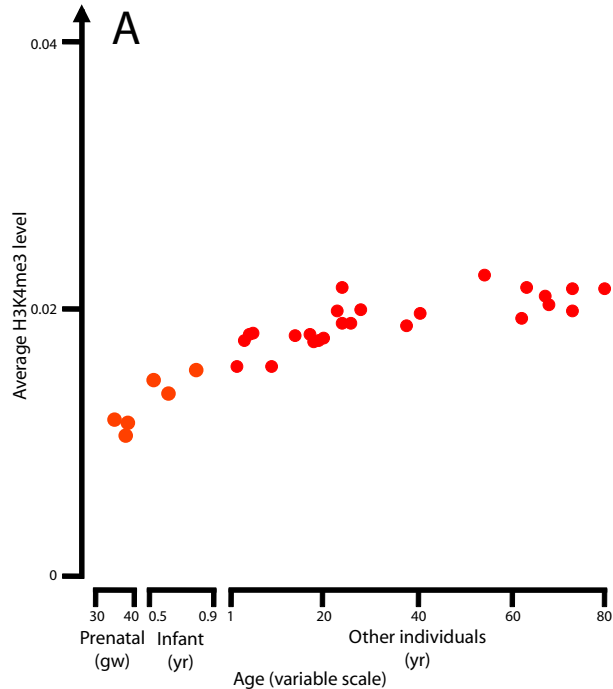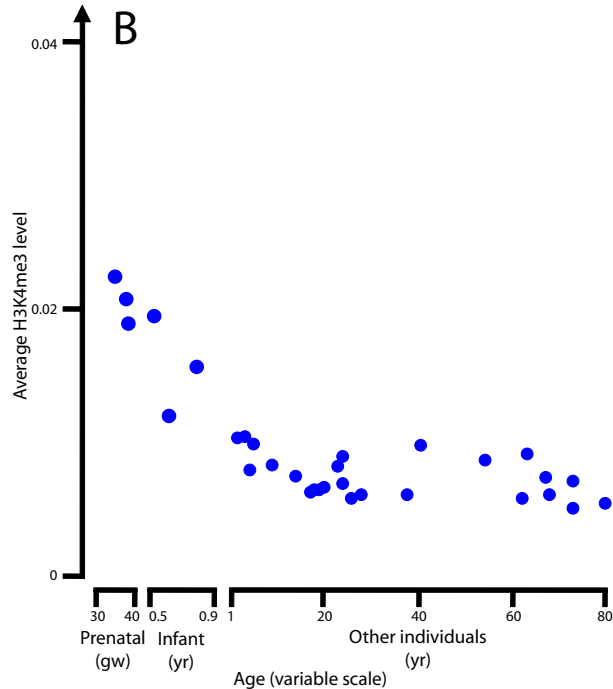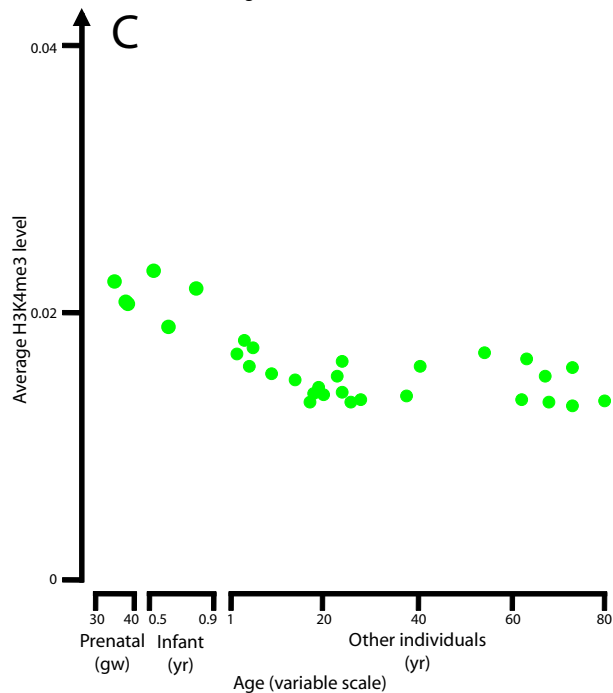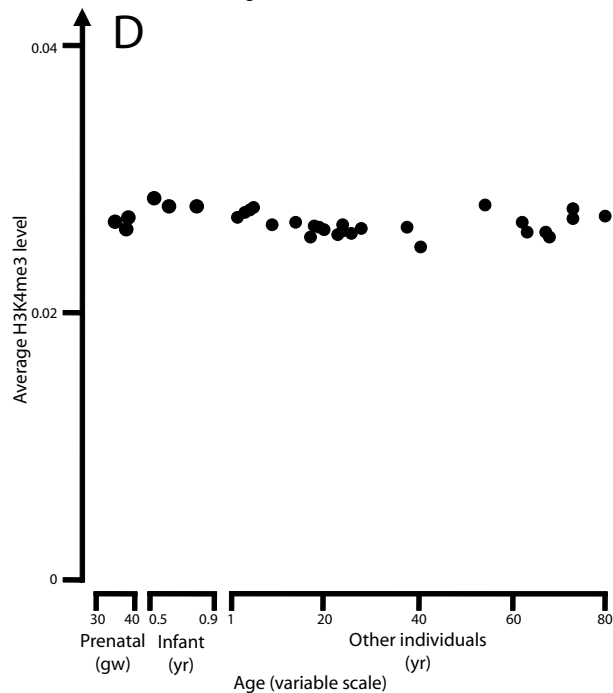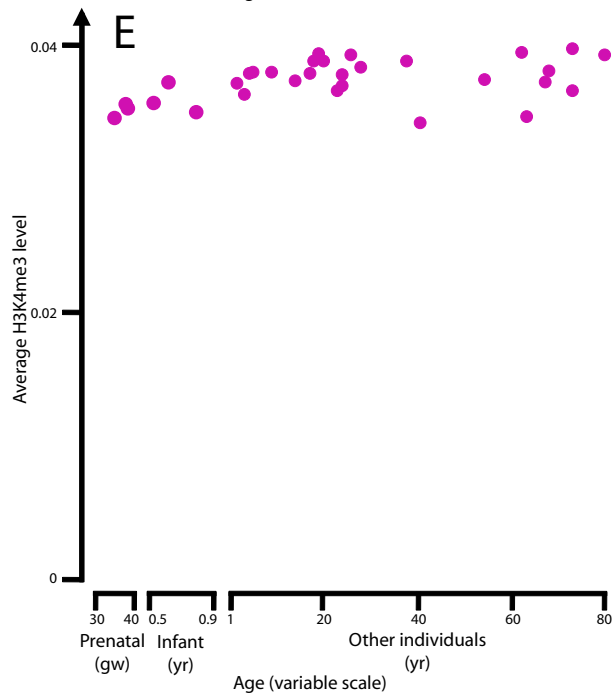

Supplement: Figure S3 — Average age profiles for H3K4me3 level for five clusters obtained by k-means clustering. (A) Cluster 1; (B) Cluster 2; (C) Cluster 3; (D) Cluster 4; (E) Cluster 5. (PDF) [file pgen.1003433.s003.pdf]

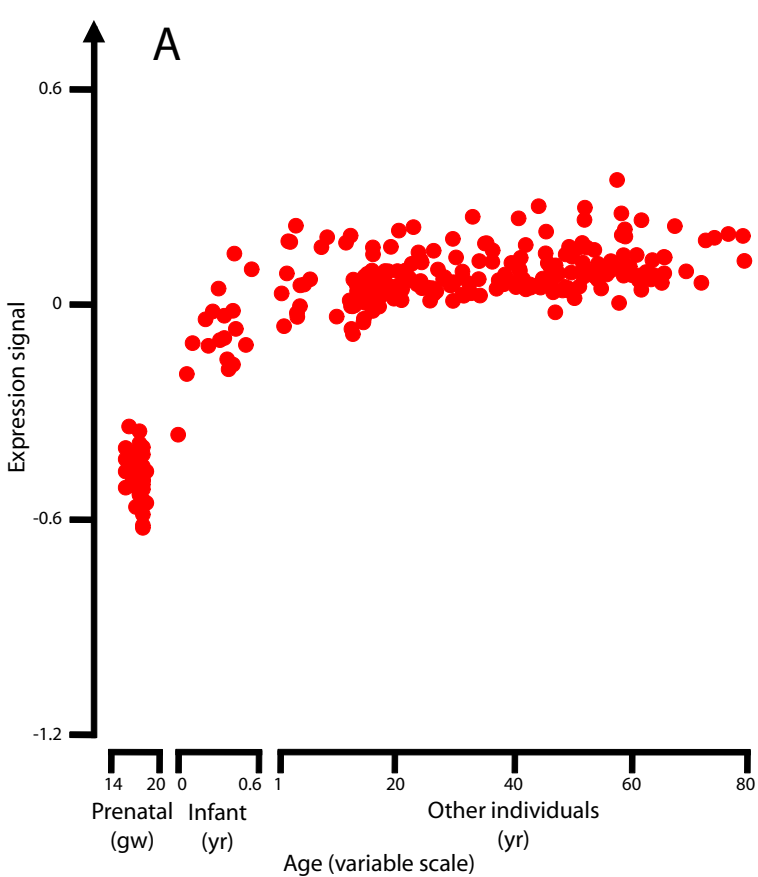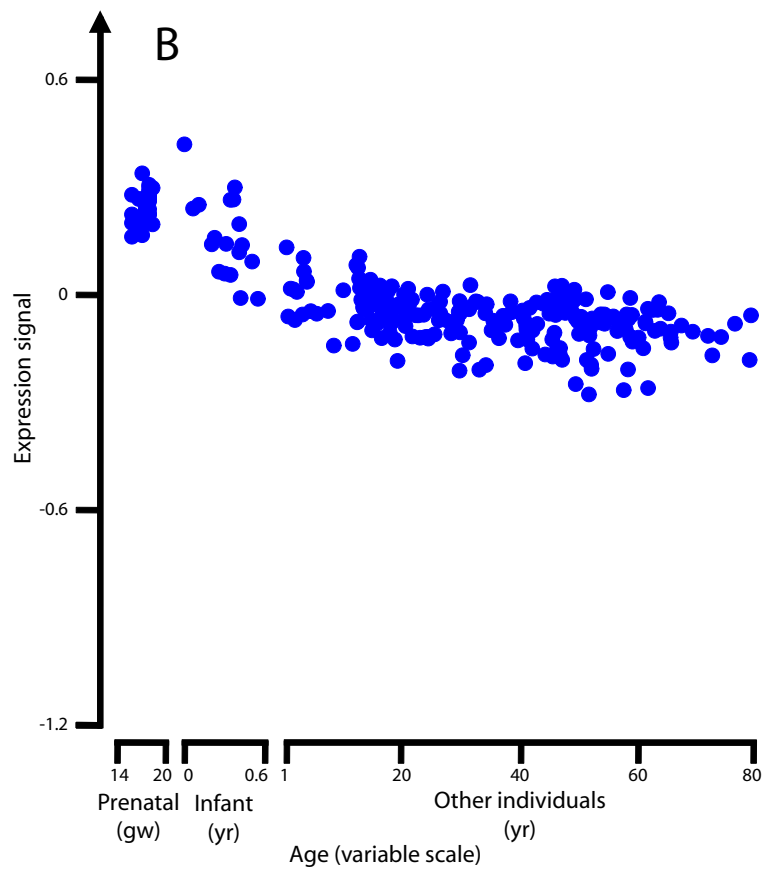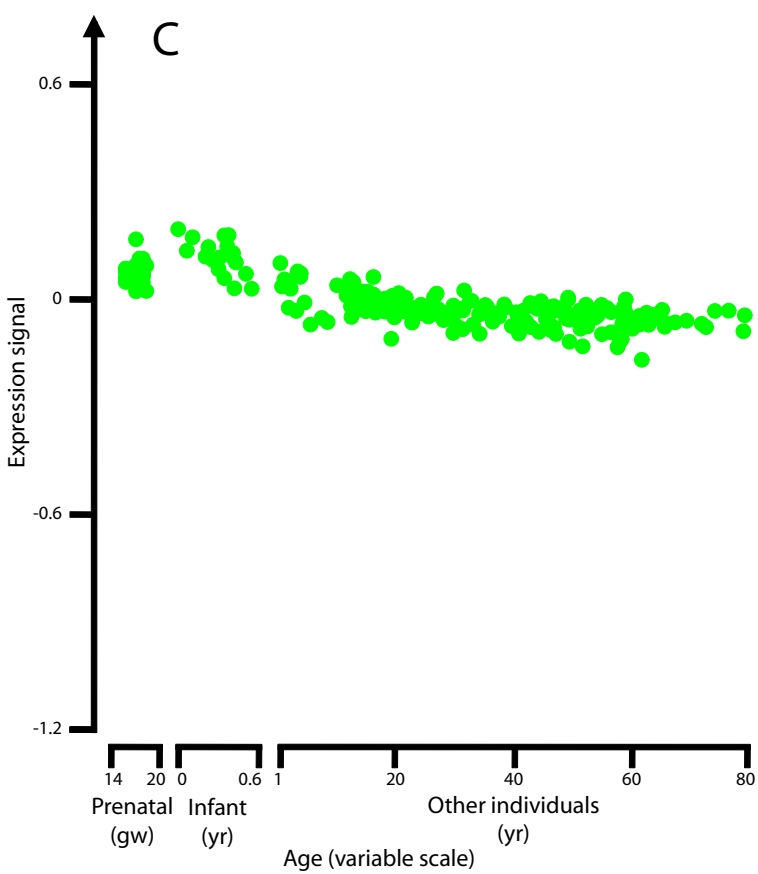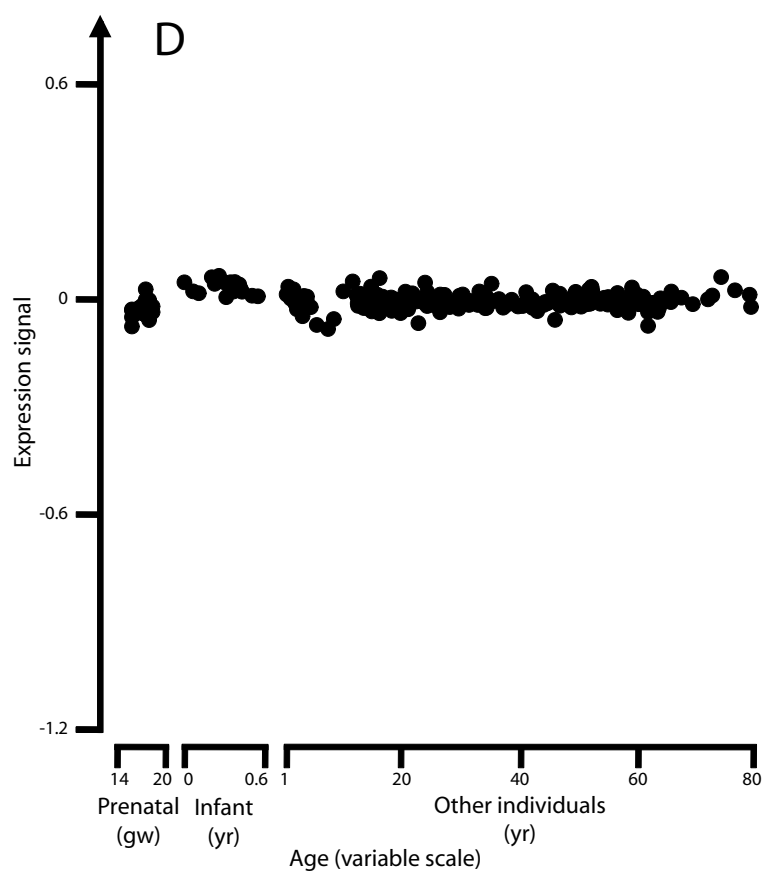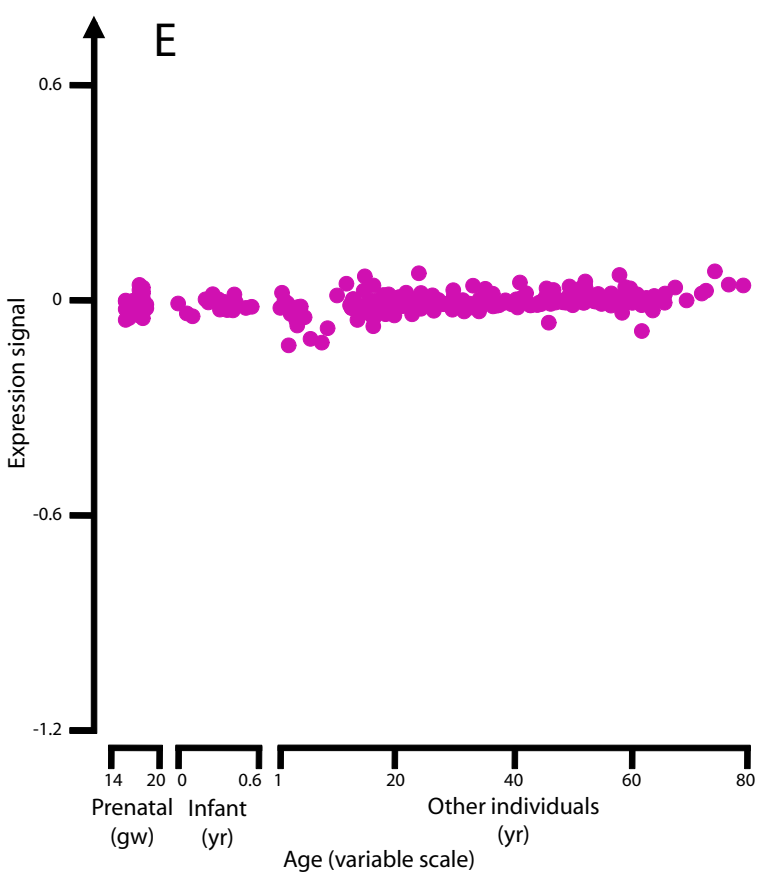

Supplement: Figure S4 — Average age profiles for expression level for five clusters obtained by k-means clustering for H3K4me3. (A) Cluster 1; (B) Cluster 2; (C) Cluster 3; (D) Cluster 4; (E) Cluster 5. (PDF) [file pgen.1003433.s004.pdf]

A

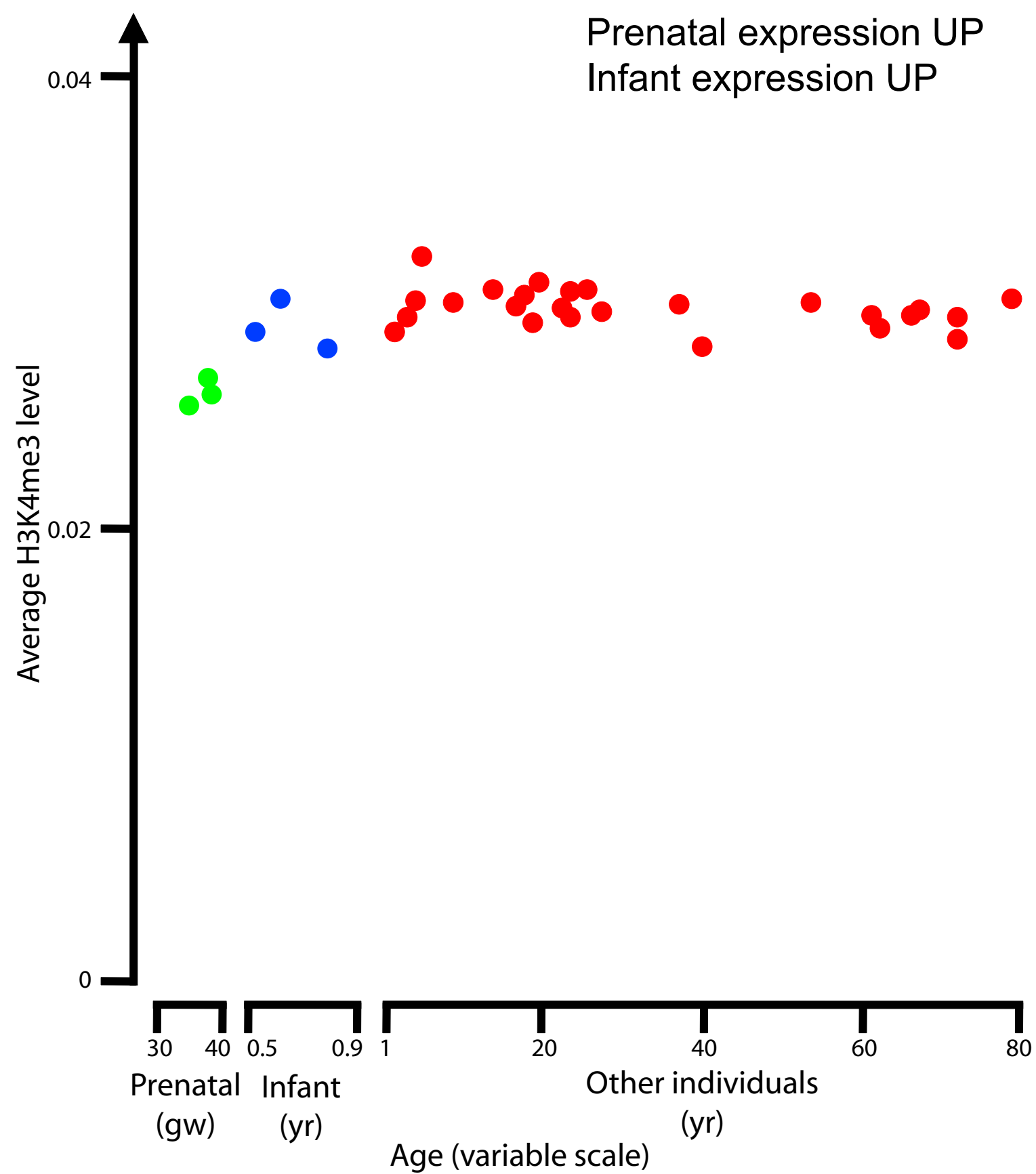

B

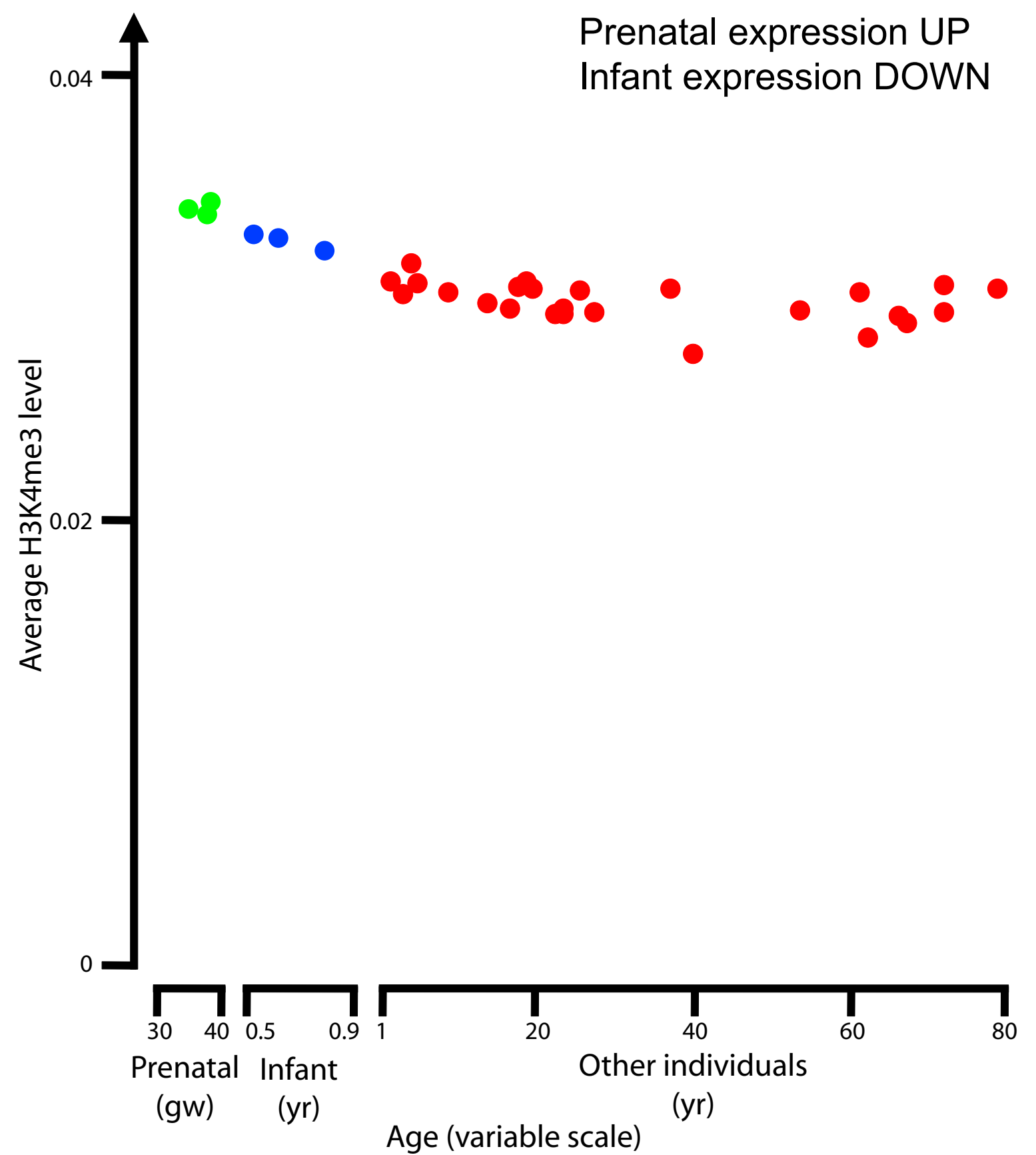

C

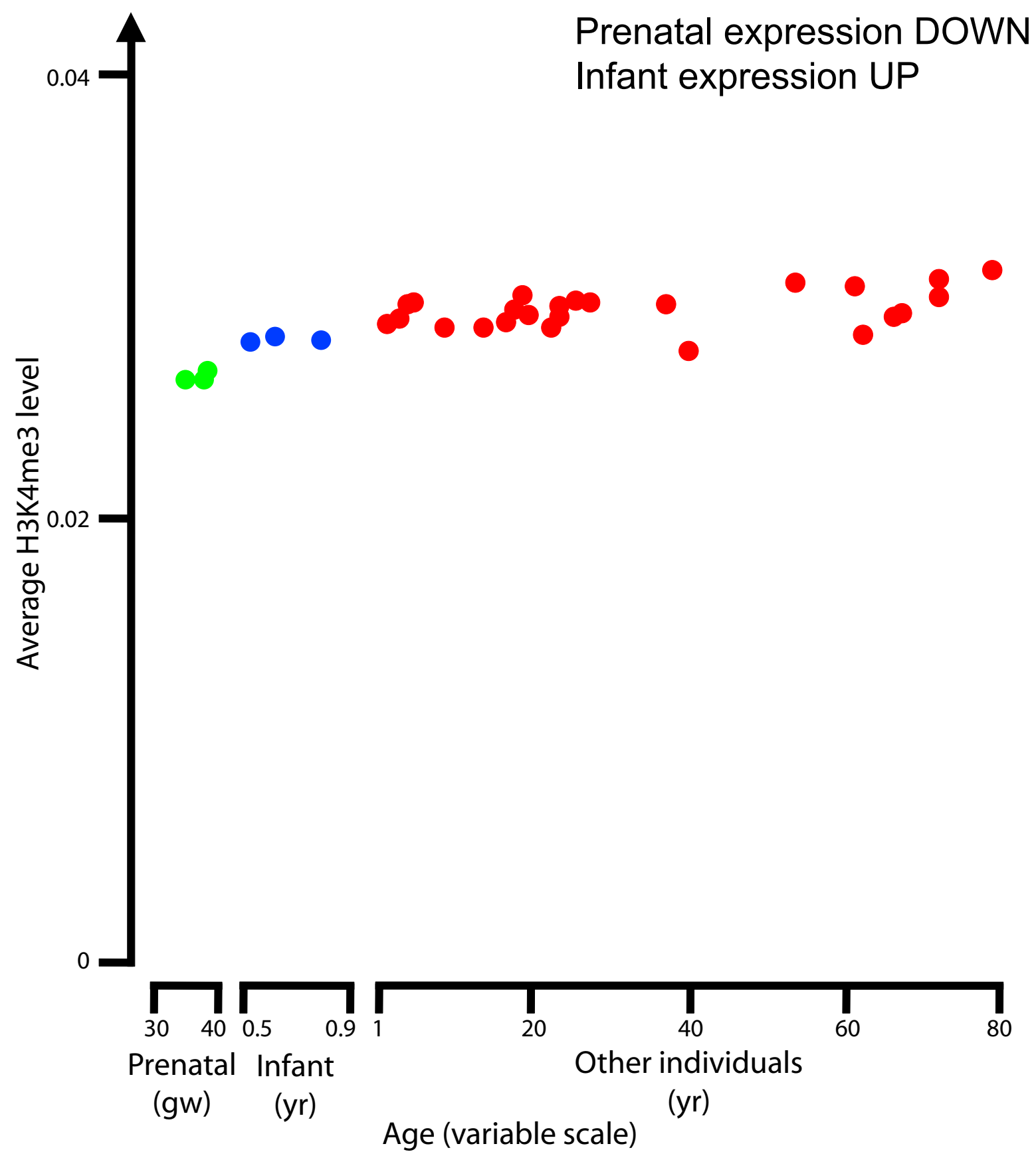

D

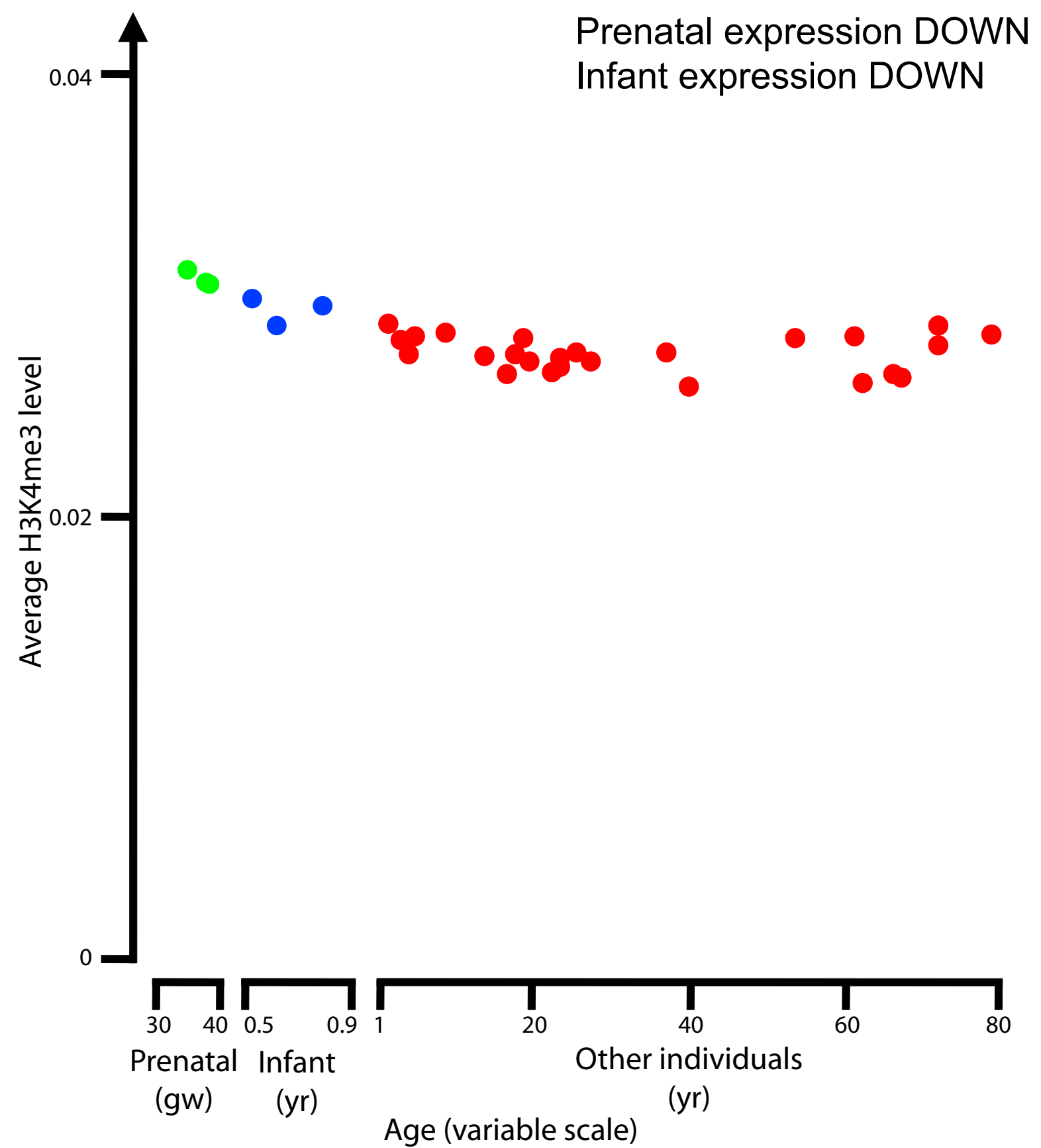

Supplement: Figure S5 — Average age profiles for H3K4me3 level for four groups of genes defined by [5]: (A) the genes that increased expression in both prenatal and infant stages (up-up genes), (B) the genes that increased expression during the prenatal stage and then decreased expression during the infant stage (up-down genes), (C) the genes that decreased expression during the prenatal stage and then increased expression during the infant stage (down-up genes), and (D) the genes that decreased expression in both stages (down-down genes). (PDF) [file pgen.1003433.s005.pdf]
